# Evolution of Tubulin Gene Arrays in Trypanosomatid parasites: genomic restructuring in *Leishmania*

**DOI:** 10.1186/1471-2164-7-261

**Published:** 2006-10-18

**Authors:** Andrew P Jackson, Sue Vaughan, Keith Gull

**Affiliations:** 1 Sir William Dunn School of Pathology, University of Oxford, South Parks Road, Oxford, OX1 3RE, UK; 2 Wellcome Trust Sanger Institute, Wellcome Trust Genome Campus, Hinxton, Cambridgeshire, CB10 1SA, UK

## Abstract

**Background:**

α- and β-tubulin are fundamental components of the eukaryotic cytoskeleton and cell division machinery. While overall tubulin expression is carefully controlled, most eukaryotes express multiple tubulin genes in specific regulatory or developmental contexts. The genomes of the human parasites *Trypanosoma brucei *and *Leishmania major *reveal that these unicellular kinetoplastids possess arrays of tandem-duplicated tubulin genes, but with differences in organisation. While *L. major *possesses monotypic α and β arrays in *trans*, an array of alternating α- and β tubulin genes occurs in *T. brucei*. Polycistronic transcription in these organisms makes the chromosomal arrangement of tubulin genes important with respect to gene expression.

**Results:**

We investigated the genomic architecture of tubulin tandem arrays among these parasites, establishing which character state is derived, and the timing of character transition. Tubulin loci in *T. brucei *and *L. major *were compared to examine the relationship between the two character states. Intergenic regions between tubulin genes were sequenced from several trypanosomatids and related, non-parasitic bodonids to identify the ancestral state. Evidence of alternating arrays was found among non-parasitic kinetoplastids and all *Trypanosoma *spp.; monotypic arrays were confirmed in all *Leishmania *spp. and close relatives.

**Conclusion:**

Alternating and monotypic tubulin arrays were found to be mutually exclusive through comparison of genome sequences. The presence of alternating gene arrays in non-parasitic kinetoplastids confirmed that separate, monotypic arrays are the derived state and evolved through genomic restructuring in the lineage leading to *Leishmania*. This fundamental reorganisation accounted for the dissimilar genomic architectures of *T. brucei *and *L. major *tubulin repertoires.

## Background

Gene expression in kinetoplastids (Euglenozoa) takes a peculiar form, quite unlike the system of promoter and terminator signals typical of other eukaryotes. Expression of several, contiguous loci occurs simultaneously through polycistronic transcription [1-3] and regulation of individual genes is largely post-transcriptional [4]. Within this context, an unusually large proportion of kinetoplastid genes is arranged in tandem gene arrays and includes transporter proteins [5], surface antigens [6] and the spliced-leader sequence responsible for cleavage of the polycistronic pre-mRNA [7]. This study addresses the evolution of α- and β-tubulin arrays in the parasitic kinetoplastids, *Trypanosoma *spp. and *Leishmania *spp., with a comparative genomic approach. These unicellular flagellates are responsible for various human diseases around the world, namely African sleeping sickness, leishmaniasis and Chagas' disease.

Tubulin is a fundamental constituent of kinetoplastid cytoskeletons, cell division machinery and motile organelles [8]. There is a large family of tubulin proteins but the α/β heterodimer is the essential building material for the microtubular cytoskeleton [9]. Trypanosomatid parasites possess an extensive array of cytoplasmic, mitotic and flagellar microtubules and recent evidence suggests that tubulin expression varies greatly during the life cycles of *Trypanosoma *and *Leishmania *spp., possibly as the relative demand for these structures fluctuates [10-14]. Indeed, in certain species, different isoforms of tubulin may have specific expression profiles related to the parasitic life cycle; for example, *L. mexicana *has a β-tubulin isoform unique to the amastigote stage found within human macrophages [15].

Kinetoplastid gene expression is based on polycistronic transcription, followed by 5' *trans*-splicing of pre-mRNA and 3' poly-adenylation to form mature transcripts [16,17]. A variety of structural motifs in untranscribed regions have been shown to regulate mRNA levels post-transcriptionally by affecting *trans*-splicing, RNA-binding capability and transcript stability [18-23]. Genes do not generally possess individual promoters and therefore, cannot be up-regulated through transcriptional initiation. Instead, where high transcript levels are required, genes may be duplicated to form tandem gene arrays, which are co-transcribed. Such dosage effects may explain why tubulin genes, and other genes, such as the paraflagellar rod proteins [24], are arrayed. However, it also seems possible for specific isoforms to be differentially expressed through regulation of particular repeats within the array, or of additional, non-arrayed (i.e., singleton) loci elsewhere in the genome [5,15,25,26].

**Figure 1:**
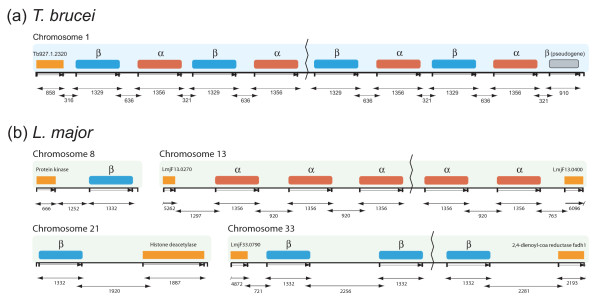
Tubulin tandem gene arrays in trypanosomatid genome sequences. a) An alternating tandem array of α- and β-tubulin repeats on chromosome 1 in *T. brucei*, showing length (in base-pairs) of coding and intergenic sequences. b) Four tubulin loci in *L. major *comprising a singleton β-tubulin locus on chromosome 8, a monotypic tandem array of α-tubulin (chromosome 33), a singleton β-tubulin locus on chromosome 21 and a β-tubulin tandem gene array (chromosome 13). Locus names refer to identifier tags in GeneDB annotations.

In yeast, equal concentrations of α and β tubulin are required for normal cellular function [27,28]. Assuming that such coordination is desirable in trypanosomatids, the precise arrangement of tubulin genes could be crucial. Recently completed genome sequences have exposed the full extent of tandem gene arrays in *Trypanosoma brucei *[29] and *Leishmania major *[30] and these are illustrated in Figure 1. Tubulin gene arrays have long been known and used to understand gene regulation in these organisms. *Trypanosoma *spp. possess a single array of alternating α- and β-tubulin genes [31-35] (see Figure 1(a)), while *Leishmania *spp. possess separate α-tubulin and β-tubulin arrays on different chromosomes [36,37] (see Figure 1(b)). The tandem repetition of these two character states suggests that they are independent solutions to a common need. Given that an alternating array is the more intuitive means of maintaining transcriptional parity between the α and β heterodimeric tubulins, quite how and why such a fundamental genomic rearrangement occurred in one or both lineages requires explanation.

This study examined the genomic architecture of tubulin tandem arrays in a range of kinetoplastids and combined this with bioinformatic analyses of various genome sequences to identify the origin of the two character states found in *Trypanosoma *spp. and *Leishmania *spp. It included representatives of the three non-parasitic clades within the Kinetoplastida, the 'bodonids', which together comprise the natural outgroup of trypanosomatids [38,39]. A phylogeny for tubulin genomic architecture was created to ask several questions: first, can the relatedness of tubulin loci be traced through comparison of their genomic positions; second, what is the polarity of the character transition, i.e., is one alternating array or two monotypic arrays the derived state; and third, if one state is derived, what is the model for the genomic rearrangement.

## Results

### Overview

The genomic arrangement of tubulin genes among kinetoplastids was examined in three stages. First, comparison of the completed genome sequences for *T. brucei *and *L. major *established the relationships between the distinct tubulin loci in these two organisms. Second, specific and degenerate polymerase chain reaction (PCR) primers, able to amplify the intergenic sequence (IGS) between any combination of α- and β-tubulin genes in tandem were designed from sequence alignments spanning the entire Kinetoplastida. These primers were used for molecular screening of various species, including the non-parasitic bodonids. Third, bioinformatic screening of draft genome sequences for tubulin loci in several other species allowed their character states to be confirmed. The characters identified by the second and third parts are described in Table 1 and were mapped on to an evolutionary tree to infer the ancestral state prior to the origins of *Trypanosoma *and *Leishmania*, and thereby, determine the phylogeny of tubulin tandem gene arrays.

#### Comparison of gene order in *T. brucei *and *L. major *genome sequences

**Figure 2:**
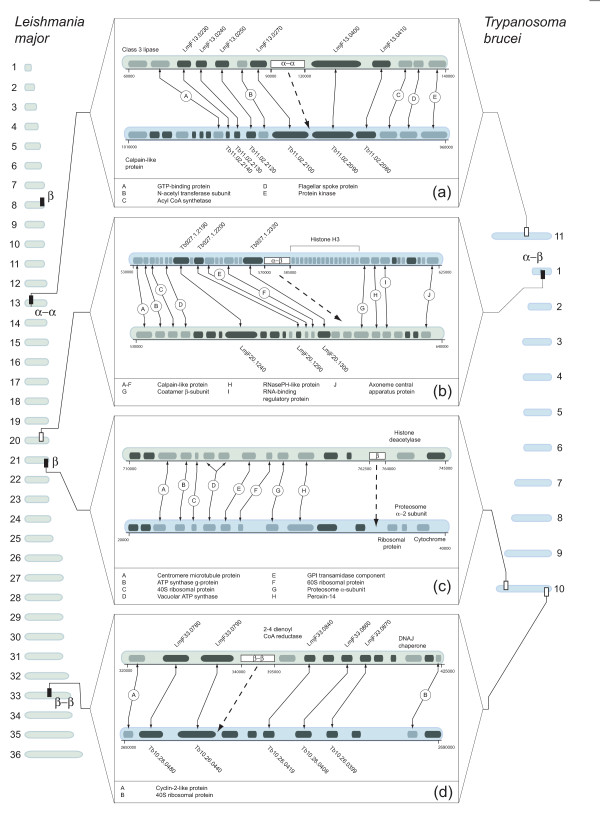
Comparison of gene order around tubulin tandem gene arrays between *T. brucei *and *L. major*. Chromosomes for each organism are represented at left (*L. major*) and right (*T. brucei*); marked are the locations of tubulin loci (filled boxes), or the conserved positions of deleted tubulin loci (open boxes). These locations are linked to a panel showing the gene order in *T. brucei *(shaded blue) and *L. major *(shaded green) in each case: (a) α-α array, (b) α-β array, (c) single β-tubulin on chromosome 21 in *L. major*; and (d) β-β array. Note that no homeologous location exists in *T. brucei *for the chromosome 8 single β in *L. major*. In each panel putative CDSs are shaded black, hypothetical CDSs in grey. Genes showing reciprocal BLAST scores above 1.0 e^-25 ^are connected by arrows. Circled letters refer to gene names where available. For hypothetical genes, the identifier tags are given next to each locus. The supposed positions of deleted tubulin loci, based on conserved synteny, are identified by dashed arrows. The scale is given in base-pairs and refers to chromosomal position.

The gene order surrounding the alternating array in *T. brucei *was conserved on chromosome 20 of *L. major*. A string of calpain-like genes, upstream in *T. brucei*, was contiguous with coatamer β-subunit and RNase-like genes, which were downstream in *T. brucei *(see Figure 2(b)). Thus, while there was a homoeologous position for the alternating array in *L. major*, there was no trace of tubulin, or the adjacent histone gene array found in *T. brucei*.

There was also colinearity around the location of the *L. major *arrays. The α-tubulin array on chromosome 13 was flanked by an N-acetyl transferase subunit gene and a long-chain fatty acid CoA ligase gene, as well as other hypothetical loci. These genes are adjacent on chromosome 11 in *T. brucei *(see Figure 2(a)), and hypothetical genes in syntenic positions showed sequence homology. Likewise for the β-tubulin array; two hypothetical genes that flank the array in *L. major *(LmjF33.0790 and LmjF33.0840) showed high BLAST scores to contiguous genes on chromsome10 in *T. brucei*: Tb10.26.0440 (7.9e^-34^) and Tb10.26.0430 (8.4e^-141^) respectively. The position of the singleton β-tubulin locus on chromosome 21 in *L. major *was partially conserved in *T. brucei*. The upstream gene order in *L. major *was recapitulated in the subtelomeric region of chromosome 10 in *T. brucei*; however, there was no tubulin locus and no conserved synteny downstream, suggesting that the tubulin locus corresponds to a strand break-point (see Figure 2(c)). No homoeologous position was identified in the *T. brucei *genome for the chromosome 8 β-tubulin locus in *L. major*.

#### Sequencing of tubulin arrays in further Kinetoplastida

Insect-parasitizing trypansomatids and non-parasitic bodonids were screened for tandem tubulin genes using specific and degenerate PCR, to provide outgroup comparisons to character states in *Trypanosoma *and *Leishmania*. Products that were amplified and sequenced from kinetoplastids without genome projects are shown in Table 1; these were non-coding IGS for the most part, although fragments of tubulin coding sequence (CDS) were present at either end to enable positive identification of gene order. *L. mexicana*, and *C. fasciculata *both possessed monotypic arrays and IGS from both α- and β-tubulin arrays were at least partially homologous with those from the existing *Leishmania *sequences. A monotypic α-tubulin gene array is also documented in *Leptomonas seymouri*, which is a close relative of *Leishmania *[36], (this information is included in Figure 4). There was no evidence of alternating arrays in any of these species. Three stercorarian trypanosomes, *T. grayi, T. pestanai *and *T. cyclops*, each possessed alternating arrays, with no evidence for monotypic arrays. While there are some gross similarities in base composition and polypurine strings, the IGSs from these species and *T. cruzi *did not align. Alternating arrays were also identified in the endosymbiont-bearing *Crithidia deanei *and another insect-parasitizing trypanosomatid, the aposymbiotic *Herpetomonas megaseliae*. Primers designed to amplify monotypic arrays produced bands for these two species when visualised on electrophoretic gels, but these were weak and inconsistent. Once sequenced, such bands from *C. deanei *were shown to result from mis-priming downstream from tubulin loci.

Table 1 includes many instances where a particular array could not be found or amplified (denoted by 'x'). This could reflect a genuine absence, as in the case of completed genome sequences, or it could reflect a failure of particular reactions, perhaps due to poor primer annealing. For instance, it was not possible to amplify the β-α IGS for *T. pestanai, C. deanei, Neobodo designis *or *Bodo saltans*; these sequences may or may not exist, for instance, *Bodo *may have only single β- and α-tubulin genes in tandem, nonetheless, their omission does not prevent the character states from being identified from the available evidence.

A variety of degenerate primer combinations generated products when applied to the non-trypanosomatid kinetoplastids, *B. saltans, Parabodo caudatus *and *N. designis*. When applied at relatively low annealing temperatures (around 52–58°C), this tended to permit priming at several sites, including on the wrong tubulin. However, this partial specificity was sufficient to amplify fragments of tubulin, which were then used to design secondary primers. These successfully generated specific products that confirmed the presence of alternating arrays in all three species. When sequenced, those products that had previously indicated monotypic arrays were shown to be IGS from the same alternating arrays and therefore, the result of mis-priming. IGSs from the three species could not be aligned.

The IGSs between tubulin repeats varied greatly in size and content, over relatively short evolutionary distances. IGSs within *Leishmania *aligned but showed substantial length variation, owing to repetitive DNA motifs. Beyond this, there was no sequence homology among IGSs of any array type. There were no obvious sequence motifs shared by IGSs, although Figure 4 shows that polypyrimidine and polypurine strings, as well as microsatellites, were very common.

#### Arrangement of tubulin genes in *Trypanosoma *genome sequences

Mining the *T. congolense *genome sequence produced two contigs that include alternating arrays of α- and β-tubulin genes, as in *T. brucei*; these can be retrieved from geneDB using the identifiers *congo819f03.q1k *and *congo_endsN14h02.p1k *respectively. The nucleotide sequence for the locus directly upstream of the array in *T. brucei *(Tb927.1.2320) was used to search *T. congolense *unassembled reads. Homologous sequences were identified and tiled together to complete the *T. congolense *homolog to the upstream locus; this showed 67% identity to the *T. brucei *gene. Tiling downstream from this gene showed that the homolog to Tb927.1.2320 is 234 bp upstream of an α-tubulin and then a further β-tubulin; it also demonstrated that IGSs following α- and β-tubulin copies are 353 bp and 443 bp in length respectively and do not align. Copies of each IGS were identical except for minor length differences in repetitive regions.

Two contigs confirmed that the situation is very similar in *T. vivax*. The first, retrievable from geneDB with the identifier *tviv499h03.p1k*, comprised an α-tubulin gene and homologs to loci directly upstream of the array in *T. brucei*, with conserved synteny. The second, identified by *tviv1885f05.p1k*, included six tubulin genes in an alternating array. The IGSs again formed two classes following α- and β-tubulin duplicates and were 332–339 bp and 492–497 bp in length respectively. There was some length variation between repeats due to repetitive elements within the IGSs.

**Figure 3:**
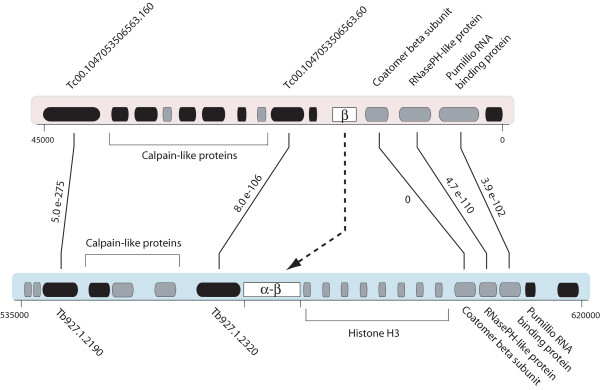
Comparison of gene order around the tubulin tandem array in *T. brucei *(shaded blue) and contig 5635 in *T. cruzi *(shaded red). Predicted CDS are shown in grey, hypothetical CDS in black. Genes with significant reciprocal BLAST matches are connected by vertical lines and labelled, showing the relevant e score. The dashed line connects the putative tubulin homologs.

No array is evident from the current release of the *T. cruzi *genome sequence [30]. However, it is known that an alternating α-β array exists [40,41], and sequence data independently deposited in GenBank comprises β- and α-tubulin genes in tandem (accessions [GenBank: AF091836] and [Genbank: M97956], among others). Otherwise, the genome sequence contained three contigs that include tubulin. The first (Tc00.1047053506563.40) showed a single β-tubulin and three other loci; together, they were colinear with chromosome 1 in *T. brucei*, and indicate that this single β-tubulin gene occupies a homoeologous position to the alternating array (shown in Figure 3). Given that an alternating arrangement is known to exist in *T. cruzi*, and is not represented elsewhere, this contig is interpreted as the location of the alternating array in *T. cruzi*. Since the array may start and end with a β-tubulin duplicate, the single β-tubulin locus could represent a 'collapsed' array. The second contig (Tc00.1047053509003.70) was a duplicate of the first, although the β-tubulin is annotated as a pseudogene and apparently lacks 700 bp from the 3' end. The third contig (Tc00.1047053411235.9) included a lone α-tubulin without any contextual information. In summary, these three *Trypanosoma *genome sequences concurred with the arrangement in *T. brucei*, showing an α-β alternating array, with conservation of surrounding gene order. IGSs following the two isotypes were always dissimilar, but each class of CDS and internal IGS were consistently concerted. There was no evidence for further tubulin loci.

#### Arrangement of tubulin genes in *Leishmania *genome sequences

The above analysis suggested that the *Trypanosoma *tubulin sequences were found only in a single alternating array. The situation in *Leishmania *spp. is more complex with multiple β-tubulin loci. The current release of the *L. infantum *genome sequence includes homologs to each *L. major *tubulin locus (shown in Figure 1). Searching with BLAST located two α-tubulin genes in tandem on contig 2963; comparison of the 958 bp IGS with *L. major *confirmed that it shows 95.5% homology to the α-array on chromosome 13. Similarly, tandem β-tubulin genes on contig 4336 had an IGS of 2217 bp, which were 94.2% homologous to those on chromosome 33 in *L. major*. Contig 4108 included a single β-tubulin and upstream loci that showed conserved synteny with chromosome 21. The 5' untranscribed region (UTR) of this single β-tubulin gene showed partial homology with the β-array (as in *L. major*) but the 3'UTR was unique. Finally, contig 4260 included a single β-tubulin gene with unique 5' and 3'UTRs that were 96% identical to those around the chromosome 8 locus in *L. major*.

At the time of writing, the draft genome sequence of *L. braziliensis *was available as a preliminary, automated assembly. This included partial α- and β-tubulin genes in approximately homoeologous positions to those in *L. major*. However, these genes did not comprise complete coding sequences and many of the surrounding loci present in *L. major *were not present in the draft assembly. In fact, the presence of homologs to each *L. major *locus was confirmed by manually assembling these loci in *L. braziliensis *by tiling reads together, using the last 100 bp of each read to locate the next. Three distinct 3' UTRs were identified by searching the read catalogue with the C-terminus of the *L. major *β-tubulin gene.

Tiling downstream from the first of these distinct sequences produced the N-terminus of another β-tubulin gene. This indicated the presence of (at least) a tandem pair of β-tubulin duplicates. It was not possible to confirm this by tiling inwards from flanking loci due to sequence gaps on both sides. The IGS between these duplicates was 1629 bp in length and showed sequence homology with the *L. major *β-β tubulin array IGS throughout its length (although its was substantially shorter due to indels).

The second distinct 3'UTR identified by BLAST also appeared to tile into another β-tubulin gene but, on closer inspection, this resulted from a section of repetitive DNA on either side of the tubulin that caused one to tile back into the N-terminus. In fact, this UTR corresponded to the chromosome 8 locus, which was confirmed by searching the read catalogue for the locus upstream of the chromosome 8 β-tubulin gene in *L. major *(LmjF08.1280). Tiling downstream from this identified a putative ortholog to LmjF08.1280 in *L. braziliensis*.

The third distinct UTR did not tile into another β-tubulin gene but a homolog to histone deacetylase gene, which was downstream of the chromosome 21 β-tubulin in *L. major*. After locating a homolog to the upstream locus in *L. major *(LmjF21.1855) and tiling downstream, the 5'UTR of a β-tubulin gene was identified; this confirmed the presence of an ortholog to the chromosome 21 locus. Tandem α-tubulin genes were identified with BLAST and shown to occupy a homoeologous position to the array in *L. major*, based on conserved synteny of surrounding loci. In summary, all three *Leishmania *genome sequences supported the presence of one α-tubulin locus on chromosome 13 and three distinct β-tubulin loci; two singletons are found on chromosomes 21 and 8, and an arrayed locus on chromosome 33. In all three species the three β-tubulin loci were flanked by distinct untranscribed regions, except the 5' UTRs of the chromosome 33 and 21 loci, which were partially homologous.

#### Phylogeny of tubulin genomic architecture

**Figure 4:**
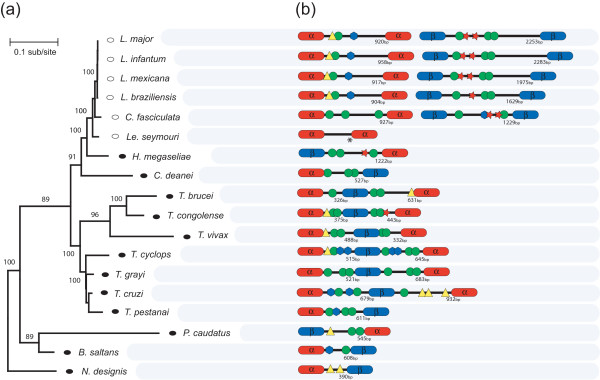
Evolution of the genomic architecture of tubulin loci in Kinetoplastida. (a) A Maximum likelihood phylogram of relationships among kinetoplastids, estimated from small subunit ribosomal RNA sequences (maximum of 2065 bp), with a GTR+I+Γ substitution model. Scale bar in substitutions per site. Values at nodes refer to non-parametric bootstrap values, out of 100. Terminal nodes are shaded according to their character state: alternating arrays (black) or monotypic arrays (white). The tree is rooted with bodonid sequences. (b) Cartoons of tubulin tandem arrays identified in kinetoplastids. Intergenic sequences are indicated with horizontal lines and include symbols referring to particular non-coding sequence motifs: polypurine tract (blue hexagon), polypyrimidine tracts (green circle), microsatellites (yellow triangle) and recognised cleavage signal (red star).

Phylogenetic estimation using 18S rRNA sequences produced a resolved and robust topology, with high bootstrap values at most nodes; the topology is consistent with previous reconstructions using this marker [38,42-45]. The rRNA phylogeny is shown in Figure 4, the genomic architecture in each species is shown alongside, as well as sequence motifs identified in intergenic sequences. The figure demonstrates that the monotypic arrays in *Leishmania *spp., *C. fasciculata *and *L. seymouri *are the derived condition and have evolved from an ancestor bearing an alternating array. This derivation occurred once and has been maintained in all *Leishmania *species inspected here. Conversely, all *Trypanosoma *spp. have maintained an alternating arrangement. Alternating arrays in all three non-trypanosomatid clades (*Bodo, Parabodo *and *Neobodo*) suggests that this transition has only occurred once in the Kinetoplastida.

## Discussion

Through a combination of molecular screening of various kinetoplastid genomes and bioinformatic screening of trypanosomatid genome sequences, the genomic organisation of tubulin genes in these protists has been identified. The alternating tubulin array in *T. brucei *and the distinct, monotypic arrays in *L. major *are representative of their respective genera and mutually exclusive, i.e., they are not modified states of a common ancestral character. Rather, two results confirm that the monotypic arrays of *Leishmania *spp. and their close relatives (*Crithidia fasciculata *and *Leptomonas seymouri*) represent a fundamental rearrangement of tubulin genes, formed *de novo *in novel genomic locations. First, the presence of alternating arrays in all three clades of non-parasitic kinetoplastids indicates that this was the character state in the ancestral trypanosomatid. Second, gene order comparisons demonstrate that, while the chromosomal locations of tubulin arrays are reciprocally conserved, the alternating array has been entirely abolished in *L. major *and *T. brucei *has no orthologs to the monotypic arrays.

An important feature of this transition is that the alternating locus has been replaced by several novel loci without any obvious links, other than the tubulin sequence itself, between the ancestral and derived character states. Comparative genomics is beginning to generate a consensus on the evolution of genomic structure, with a distinction drawn between chromosomal rearrangements that cause disruptions in macrosynteny and may affect karyotype, and smaller, segmental duplications, inversions and transpositions, (often accompanied by differential gene loss), which all disrupt microsynteny [46]. These events are taxonomically widespread and responsible for the creation of novel genes [47,48]; segmental duplication and subsequent gene loss largely determines gene order evolution in comparisons of yeast genomes [49] and in primates [50], while gene order in *Drosophila *spp. is greatly affected by paracentric inversions [51]. In addition to these mechanisms, selfish elements have been frequently implicated in the translocation of genomic DNA, for example in most plants [52] and in trypanosomes [53].

In this case coding sequences have moved to new locations that otherwise retain colinearity between *T. brucei *and *L. major*. The mechanism of translocation is unclear since nothing else that may have transposed simultaneously has survived, i.e., no neighbouring genes of the ancestral locus are seen at the derived loci to provide evidence for their source. The presence of 'calpain-like' proteins upstream of the alternating locus in *T. brucei *might suggest that this locus could 'hitch-hike' to new locations as a result of ectopic recombination between members of the calpain-like gene family; but the absence of calpain-like proteins adjacent to any derived loci precludes this. Breakpoints have been identified in otherwise excellent synteny between trypanosomatid genomes [53]; it emerged that breakpoints coincide with retrotransposon hotspots, suggesting that changes to gene order are mediated by selfish elements known from these organisms. A non-autonomous retroelement (RIME) sequence [54] does occur at the end of the alternating array in *T. brucei*, but this seemed to be associated with the movement of a contiguous array of histone H3 genes rather than the tubulin genes, as the RIME and histones were not found in other species where gene order was otherwise conserved. The two singleton loci in *Leishmania *did occur at the extreme 5' end of subtelomeric regions, where retrotransposons are frequently found [52-54], but beyond this there is no evidence for the role of selfish elements. The relationships of tubulin loci to strand-switches may provide evidence of previous chromosomal rearrangements, or place tubulin loci in regions of frequent rearrangement. The α-β array is 40 kb downstream of the nearest strand-switch. In *L. major*, the α-α array and chromosome 21 β-tubulin are only 25 Kb from a strand-switch, but for the β-β array and chromosome 8 β-tubulin, the distances are 75 Kb and 60 Kb respectively [55]. Hence, the significance of chromosomal rearrangements may yet become clear, but the proximity of strand-switches does not currently look unusual, as they are relatively frequent in these genomes.

Hence, the physical mechanism responsible for new tubulin loci is unclear. Clearly, segmental duplications caused tubulin genes to be translocated from the ancestral locus around the genome; this must have created some kind of transitional structure in which both original and novel loci coexisted, before the ancestral locus was abolished. A future study should seek evidence in appropriate non-parasitic relatives of *Leishmania*, such as *Herpetomonas *spp.,*Crithidia *spp. and *Phytomonas *spp.; these organisms are the closest relatives to *Leishmania *still retaining alternating arrays; they could provide a transitional state between alternating and monotypic character states, if they possess other tubulin loci, perhaps additional arrays or lone genes, and if the gene order around these additional features could be related to loci in *Trypanosoma*. In other words, these organisms may retain elements of the ancestral, transitional character states in a manner expressly not seen in *Leishmania*. Genome structures diverge faster than genome sequences due to a higher rate of segmental duplication [46]; this suggests that most duplications are removed through purifying selection but also that there are regular opportunities for new loci. The issue here is, regardless of how tubulin loci were duplicated, why the selective environment changed from purifying to promoting their establishment.

Given the likely difficulties ensuing from a monomer production imbalance in a polymer/dimer polymerisation equation it is understandable that cells have developed transcriptional and post-transcriptional controls to regulate equivalent amounts of α and β tubulin [56]. Coupled with the fact that the trypanosome requires large amounts of tubulin dimer, the *T. brucei *arrangement of a large number of alternating genes provides a very reasonable solution in an organism lacking transcriptional controls. Thus, what drivers might cause *Leishmania *to discard this structure? The first move appears to have been to separate the α and β tubulin loci into new sites. If large and essentially equivalent gene numbers are maintained at these sites and transcriptional passage is similar then this might appear to offer little disadvantage to the organism. At this time, we suggest that the alternating isoforms were separated to allow differential expression, and perhaps unilateral changes in regulation, of α- or β-tubulin or both. Within the context of polycistronic transcription, this would be the benefit of physical transposition, but the need that this transition fulfilled is not known.

Certainly, the evolution of differential expression cannot be related to the evolution of new life stages, since the major lifecycle difference influencing the cytoskeleton between *T. brucei *and *L. major *is that the latter produces an amastigote form that lacks a motile flagellum. However, an amastigote is also formed by *T. cruzi*, which has conserved the single array; furthermore, it is clear that the monotypic arrays evolved before the amastigote phase in *Leishmania *(as they are present in *C. fasciculata *and *L. seymouri *also). Equally, the derivation of monotypic arrays cannot be related to the evolution of additional, singleton loci on chromosomes 8 and 21 in *Leishmania *spp. [57], since these too evolved in *Leishmania*, after the monotypic arrays. Separation of the tubulin isoforms may have facilitated novel β-tubulin loci but it cannot have been derived from the same fundamental causes.

## Conclusion

The restructuring of tubulin repertoire in trypanosomatids is an example of a very stable system being rapidly and entirely replaced by a novel derivation. The evolutionary causes of the replacement of an alternating α-β tubulin tandem array by separate, monotypic arrays probably reflects new expression regimes that became apparent in the lineage leading to *Leishmania*, and segmental transpositions that gave the opportunity to craft new loci. The role of transposition in the evolution of tubulin repertoire may itself reflect a ubiquitous constraint in kinetoplastids, and the reason why tubulin tandem arrays exist at all, the absence of individual gene promoters. The arrangement of tubulin genes in arrays ensures high expression levels in the context of polycistronic transcription, but prevents the divergence of non-coding regions and functional specialisation, probably due to repeated crossing-over between duplicate alleles and, consequentially, concerted evolution [58]. Setting aside the exact reasons why new tubulin loci were established, transposition events in the ancestor of *Leishmania *may have been essential to overcome the historical constraint inherited from non-parasitic kinetoplastids, and facilitate the evolution of novel expression patterns. Furthermore, it was observed here that genomic environments around tubulin loci, past and present, are widely conserved across species while tubulin genes themselves are not. This suggests that, once transposed, these new loci supplanted the original locus, leading to its rapid eradication.

## Methods

This study utilised the completed and draft genome sequences of various parasitic kinetoplastids: *Trypanosoma brucei, T. cruzi, T. congolense, T. vivax, Leishmania major, L. infantum *and *L. braziliensis*. These supplemented the molecular screening of related species that required cell culture (see references for culture details): *T. pestanai, T. grayi *and *T. cyclops *[45,59],*Herpetomonas megaseliae, Crithidia fasciculata, C. deanei, Bodo saltans *(JC02 strain, [39]), *Parabodo caudatus *and *Neobodo designis *(Longstock strain, [39]). The completed genome sequences of *T. brucei *and *L. major *were first compared to establish the relationships (if any) of the genomic locations where tubulin loci now reside. Various other parasitic trypanosomatids and free-living bodonids were cultured and screened using specific and degenerate PCR to establish their character states. Draft genome sequences were interrogated for tubulin loci and to score each species for its character state (i.e., alternating or monotypic array).

### Comparing genomic location and gene synteny

The genomic location of the *T. brucei *array is specified by gene order. This site was identified in *L. major *to establish if the location was conserved through time or, alternatively, if tubulin rearrangements had evolved in new chromosomal environments. DNA sequences of up- and downstream coding loci were used as queries for BLAST searches of the *L. major *genome in GeneDB. Similarly, the genomic locations of all four *L. major *tubulin genes were located in the *T. brucei *genome. Comparisons were also made between *T. brucei *and *T. cruzi *to establish if both species share a homologous alternating array, on the basis of chromosomal location. *T. brucei *sequences for flanking loci were used to search among the partially assembled *T. cruzi *contigs and locate their closest homologs as before.

### Character scoring from genomic DNA

Where sequence data was gathered *de novo*, this required cell culture, genomic DNA extraction, amplification by PCR, molecular cloning and sequencing. Cell culture was carried out at room temperature for free-living species (*B. saltans, P. caudatus *and *N. designis*) in soil-extract solution, enriched with 0.25% beef extract. Parasitic species were cultured in standard media at 37°C: Warren medium ([60], for *C. deanei *and *C. fasciculata*), SDM-79 ([61], for *T. cyclops, T. grayi *and *T. pestanai*) and liver infusion tryptose ([62], for *H. megaseliae*). Genomic DNA was prepared from 50–100 ml of liquid culture (cell density: 1 × 10^6^–10^7 ^ml^-1^) by phenol-chloroform extraction and resuspension in 10 mM Tris [63].

Both specific and degenerate primers were designed to anchor within the termini of tubulin CDSs at conserved points identified from alignments of kinetoplastid α- and β-tubulin genes, which included a natural outgroup *Euglena gracilis *[64], ([GenBank: AF182555, GenBank: AF182557]). Therefore, these primers could amplify across the IGS of any potential array. Specific primers were labelled: 'sensealpha' (GAGAAGGACTACGAGGAGGT); 'antialpha' (GAGTACCAGCAGTACCAG); 'sensebeta' (CA(TC)TGGTACGTCGGATGAGGG) and 'antibeta' (GGTGG(CT)ACTGGTCT(CT)). Degenerate primers were labelled: 'alphaF' (TCGA(CT)(CT)T(GCT)ATGTAC(AC)(GC)CAAGCG); 'alphaR' (TCCTTGCC(AG)(GC)(AT)(GC)A(CT)CAGCTGC); 'betaF' (AC(GCT)G(GCT)(GCT)ATGTTCCG(CT)CGCAAG) and 'betaR' (CCAG(AT)CTG(AGT)CCAAA(GT)A(CT)(AG)AAGTTG).

In combination, these primer pairs could amplify across the IGS of α-α, β-β, β-α and α-β tandem gene pairs in any of the cultured species. All combinations of specific and degenerate primers respectively were applied to each DNA preparation. 'Touchdown' PCR was performed under the following conditions: denaturation at 95°C, extension at 70°C and annealing at 58°C for 5 cycles, 56°C for 5 cycles and 52°C for 25 cycles (for specific primary primers) or 68°C for 5 cycles, 64°C for 5 cycles and 60°C for 25 cycles (for degenerate primary primers). Products were cloned into pGEM T-easy plasmid vectors (Promega), purified from bacterial culture and sequenced using an ABI 377 automated sequencer.

### Character scoring from genome sequences: contig assembly and inspection

Tubulin genes were identified for *Trypanosoma congolense *and *T. vivax *by BLAST searching [65] within their draft genome sequences, available from the GeneDB website (Sanger Institute Pathogen Sequencing Unit [66]). *T. brucei *α- and β-tubulin DNA sequences were used as the search query. Positive contigs were then inspected using Artemis v5.0 [67] to establish if arrayed tubulin genes could be found on a single contig. At this time, the *Leishmania infantum *and *L. braziliensis *genomes were available as first draft assemblies, without any manual annotation or checking of assembly. Prior to manual revision, a preliminary assembly can make errors, especially regarding duplicate gene loci. For this reason, it was necessary for these species to identify matches to tubulin by BLAST searching among read catalogues, then use these matches to search for overlapping reads and finally to assemble contigs by tiling together individual reads. *L. major *α- and β-tubulin DNA sequences were used as the search query.

### Estimating species phylogenies

A comparative approach to the evolution of genomic characters requires a species phylogeny. This was obtained through phylogenetic analysis of small subunit ribosomal RNA sequences for the species concerned. These were selected from depositions to GenBank: *T. cruzi *[GenBank: AF232214], *T. pestanai *[GenBank: AJ009159], *T. congolense *[GenBank: AJ223563], *T. brucei *[GenBank: AJ009142], *T. grayi *[GenBank: AJ005278], T. *cyclops *[GenBank: AJ131958], *T. vivax *[GenBank: U22316], *L. major *[GenBank: X53915], *L. mexicana *[GenBank: X53912], *H. megaseliae *[GenBank: U01014], *C. fasciculata *[GenBank: Y00055], *L. infantum *[GenBank: X07773], *L. braziliensis *[GenBank: M80292], *C. oncopelti *[GenBank: AF038025], *B. saltans *[GenBank: AF208889], *N. designis *[GenBank: AY998646], *P. caudatus *[GenBank: AY490218] and *L. seymouri *[GenBank: AF153040]. Note that *C. oncopelti*, a close relative of *C. deanei*, was used as a surrogate in the rRNA alignment, given the absence of any SSU rRNA sequence for *C. deanei*. Sequences were aligned by eye and maximum likelihood phylogenetic estimation was carried out using PHYML [68,69]. A general-time reversible (GTR, [70]) model was applied, with six rate categories estimated from the data. An initial tree topology was selected through neighbour-joining. Corrections were made for both invariant sites and rate heterogeneity by estimating the proportion of invariant sites and the gamma distribution parameter (α) from the data. 100 non-parametric bootstrapped data sets were estimated.

## List of abbreviations

PCR Polymerase chain reaction

BLAST Basic local alignment search tool

GTR General-time reversible

CDS Coding sequence

IGS Intergenic sequence

UTR Untranscribed region

## Authors' contributions

APJ carried out cell culture, DNA preparation and molecular screening of kinetoplastids, as well as bioinformatic comparisons of genome sequences and drafting of the manuscript. SV produced preliminary bioinformatic analyses and gave assistance in experimental design and manuscript preparation. KG supervised the study design, concept and execution, and contributed to manuscript preparation. All authors read and approved the final manuscript.

## Figures and Tables

**Table 1:** Intergenic sequences from tubulin tandem gene arrays in kinetoplastids

	Species	Tandem gene array^b^:											
		Monotypic:						Alternating:					
		α-α			β-β			α-β			β-α		
		size	primers	accession^a^	size	primers	accession^a^	Size	primers	accession^a^	size/bp	primers	accession^a^
Genome	*Trypanosoma brucei*	X			x			326	-	Tb927.1.2340	631	-	Tb927.1.2330
	*Trypanosoma congolense*	X			x			357	-	congo819f03.q1k	443	-	congo819f03.q1k
	*Trypanosoma cruzi*	X			x			679	-	AF091836	932	-	M97956
	*Trypanosoma vivax*	X			x			488	-	tviv1885f05.p1k	332	-	tviv1885f05.p1k
	*Leishmania major*	920	-	LmjF13.0280	2253	-	LmjF33.0792	x			x		
	*Leishmania infantum*	958	-	LinJ13.0260	2283	-	contig4336	x			x		
	*Leishmania braziliensis*	904	-	-	1629	-	-	x			x		
PCR	*Trypanosoma cyclops*	X			x			515	betaF/alphaR	DQ450538	645	betaF/alphaR	DQ450538
	*Trypanosoma grayi*	X			x			521	betaF/alphaR	DQ450539	683	betaF/alphaR	DQ450539
	*Trypanosoma pestanai*	X			x			611	senseα/antiβ	DQ450540	x		
	*Crithidia deanei*	X			x			527	betaF/betaR	DQ450532	x		
	*Bodo saltans*	X			x			608	senseα/antiβ	DQ450531	x		
	*Parabodo caudatus*	X			x			x			545	senseβ/antiα	DQ450537
	*Neobodo designis*	X			x			390	senseα/antiβ	DQ450535	x		
	*Herpetomonas megaseliae*	X			x			x			1222	senseβ/antiα	DQ450536
	*Leishmania mexicana*	917	senseα/antiα	DQ450541	1975	senseβ/antiβ	DQ450542	x			x		
	*Crithidia fasciculata*	927	senseα/antiα	DQ450533	1229	senseβ/antiβ	DQ450534	x			x		

^a^Accession numbers given for completed or draft genome sequences correspond to identifier tags in genome sequence browsers [66], except for *T. cruzi*. Accession numbers given for PCR sequences and *T. cruzi *correspond to GenBank accession numbers. Contigs were manually assembled for *L. braziliensis*, therefore no identifiers are available.

^b ^'x' denotes an absence of any evidence for a given intergenic sequence, caused either by a failed reaction or a genuine absence (see text).
